# Anhedonia in Postpartum Depression: The Role of Neuroinflammation and Dopamine Dysfunction

**DOI:** 10.1016/j.bpsgos.2026.100826

**Published:** 2026-09-10

**Authors:** Julijus Motiejūnas, Jurgita Ušinskienė, Dainius Characiejus, Algirdas Tamošiūnas, Janina Tutkuvienė, Dalius Jatužis, Nerijus Masiulis, Oron Levin, Romualdas Griškevičius, Alvydas Navickas

**Affiliations:** a Clinic of Psychiatry, Institute of Clinical Medicine, Medical Faculty, Vilnius University, Vilnius, Lithuania; b Department of Rehabilitation, Physical and Sports Medicine, Faculty of Medicine, Institute of Health Sciences, Vilnius University, Vilnius, Lithuania; c Department of Radiology, Nuclear Medicine, and Medical Physics, Faculty of Medicine, Vilnius University, Vilnius, Lithuania; d Department of Pathology and Forensic Medicine, Institute of Biomedical Sciences, Faculty of Medicine, Vilnius University, Vilnius, Lithuania; e State Research Institute, Centre for Innovative Medicine, Vilnius, Lithuania; f Faculty of Medicine, Institute of Biomedical Sciences, Vilnius, Lithuania; g Department of Anatomy, Histology and Anthropology, Institute of Biomedical Sciences, Faculty of Medicine, Vilnius University, Vilnius, Lithuania; h Clinic of Neurology and Neurosurgery, Institute of Clinical Medicine, Faculty of Medicine, Vilnius University, Vilnius, Lithuania; i Department of Health Promotion and Rehabilitation, Lithuanian Sports University, Kaunas, Lithuania; j Biomedical Magnetic Resonance Imaging Unit, Department of Imaging and Pathology, Group Biomedical Sciences, KU Leuven, Leuven, Belgium

**Keywords:** Anhedonia, Dorsolateral prefrontal cortex, Inflammation, Nucleus accumbens, Postpartum depression

## Abstract

Anhedonia is a core symptom of postpartum depression (PPD); however, the underlying neurobiological mechanisms remain poorly understood. Emerging evidence suggests that postpartum biological adaptations, including sleep disruption, stress, hormonal fluctuations, and immune remodeling, may influence neural systems involved in motivation. In this review, we examine neuroinflammation as a potential contributor to maternal anhedonia through its effects on dopamine-dependent reward circuitry. We propose 2 complementary mechanisms by which inflammatory signaling may disrupt motivation: 1) local inflammatory effects within nucleus accumbens microcircuits regulating dopamine release and 2) dysfunction of corticostriatal networks, whereby inflammation impairs prefrontal regulation and reward valuation processes, leading to downstream anhedonia. Furthermore, we review neuroimaging evidence supporting reward system abnormalities in PPD and discuss how multimodal imaging approaches may help distinguish between proposed mechanistic models. Understanding these pathways may facilitate the development of biologically informed interventions targeting maternal anhedonia.

## Plain Language Summary

Maternal anhedonia is a clinically important but poorly understood feature of postpartum depression. This review examines neuroinflammation as a potential mechanism linking postpartum biological adaptations—including sleep disruption, stress, hormonal fluctuations, and immune remodeling—to the dysfunction of dopamine-dependent reward circuitry. We propose 2 complementary pathways: direct inflammatory effects within nucleus accumbens microcircuits regulating dopamine release and disruption of corticostriatal networks involved in reward valuation and motivational control. Furthermore, we evaluate neuroimaging evidence and discuss how multimodal approaches may help distinguish between these competing mechanistic models.

Postpartum depression (PPD) is one of the most common mental health conditions after childbirth. It can substantially affect maternal well-being, functioning, and the developing mother-infant relationship (1,2). The postpartum period normally involves a profound shift in motivational salience toward infant-related cues, supporting maternal responsiveness and caregiving behavior (3,4). In PPD, this adaptation may be blunted, manifesting as diminished responsiveness to infant cues and reduced motivation to engage in maternal behaviors. Understanding why maternal motivation becomes disrupted may therefore provide important insight into the neurobiology of PPD.

Pregnancy, delivery, and early postpartum recovery cause changes in immune activity, reproductive hormones, stress, and sleep patterns. Although most women experience these changes, in susceptible mothers, they increase neuroimmune dysregulation (5, 6, 7, 8, 9, 10, 11, 12, 13, 14). Inflammatory signaling may represent one pathway through which postpartum biological changes influence brain systems involved in motivation and reward.

A central candidate mechanism is the disruption of dopamine-dependent reward circuitry. Dopamine signaling within the ventral striatum (VS), particularly the nucleus accumbens (NAc), plays a critical role in motivated behavior (15, 16, 17, 18). Animal models of PPD show reduced mesolimbic dopamine activity accompanied by impaired maternal behavior and motivational deficits, whereas human neuroimaging studies report blunted neural responses to infant-related cues in mothers experiencing depressive symptoms (19, 20, 21, 22, 23, 24).

How inflammation disrupts dopamine-dependent motivation remains uncertain. Inflammatory processes may act directly within NAc microcircuits that regulate dopamine release. Alternatively, inflammation may impair distributed corticostriatal networks involving the dorsolateral prefrontal cortex (dlPFC), ventromedial PFC (vmPFC), anterior cingulate cortex (ACC), subgenual ACC (sgACC), and VS. This review examines these complementary pathways and considers how postpartum biological adaptations may converge on dopamine dysfunction to contribute to maternal anhedonia.

## Postpartum Biological Transition and Vulnerability

The postpartum period is characterized by rapid biological and behavioral adaptations. Evidence suggests that postpartum alterations in sleep quality, stress, reproductive hormones, and immune activity are associated with an increased risk of PPD and may influence neural systems involved in motivation and reward processing. These factors are particularly relevant to the present model because each can interact with inflammatory and dopamine-dependent pathways implicated in anhedonia.

### Sleep Disruption

Sleep disruption is one of the most common challenges of the postpartum period. Repeated awakenings result in fragmented sleep, reduced overall sleep duration, and restorative sleep quality (25,26). Consequently, postpartum women frequently experience fatigue, impaired cognitive performance, and reduced emotional resilience (25,27).

Sleep disturbance is a consistently identified risk factor for PPD. Poor sleep quality during pregnancy and the postpartum period has been associated with increased depressive symptoms and a greater likelihood of developing PPD (28,29). Experimental sleep deprivation in nonpostpartum populations alters hypothalamic-pituitary-adrenal (HPA) axis function and prefrontal and ventral striatal responses during reward processing, while broader evidence links sleep disturbance with increased inflammatory activity (30, 31, 32, 33). Postpartum sleep fragmentation may increase vulnerability to maternal anhedonia by simultaneously promoting inflammation and perturbing neural systems involved in reward valuation and motivated behavior.

### Hormonal Changes

Pregnancy causes increases in reproductive hormones, particularly estradiol and progesterone. These hormones decline rapidly following delivery after placental expulsion (34, 35, 36). The onset of PPD symptoms could coincide with this endocrine transition; therefore, hormonal withdrawal has long been considered a potential contributor to postpartum mood disorders.

Some women may be particularly sensitive to reproductive hormone fluctuations. Bloch *et al.* (37) demonstrated that experimentally induced estradiol and progesterone withdrawal provoked depressive symptoms in women with a history of PPD but not in healthy control participants. Attention has also focused on allopregnanolone, a progesterone-derived neuroactive steroid that modulates GABA_A_ (gamma-aminobutyric acid A) receptor function and declines rapidly after childbirth. The antidepressant efficacy of brexanolone, a synthetic form of allopregnanolone, further supports a role for hormone-sensitive neurobiological mechanisms in PPD (35,38, 39, 40).

Reproductive hormones interact directly with neural systems central to motivation. Rodent studies demonstrate that estradiol modulates dopamine signaling within the striatum and NAc, including rapid effects on dopamine release and increased motivated behavior (41,42). Importantly, parturition-associated hormonal states alter NAc dopamine responses to offspring cues: postpartum rats and ovariectomized females receiving parturient-like hormonal treatment show enhanced pup-evoked dopamine release in the NAc shell compared with nonprimed females (43,44). These findings suggest that reproductive hormonal adaptations can tune mesolimbic dopamine circuitry toward increased motivational salience of offspring. The rapid hormonal changes after delivery could reduce responsiveness to infant-related cues in susceptible mothers.

Despite this evidence, reproductive hormones alone do not adequately explain PPD. Studies have generally failed to identify consistent differences in hormone concentrations between depressed and nondepressed mothers (36,45,46). Hormonal withdrawal may contribute to a broader postpartum vulnerability in which reward circuitry plays a substantial role.

### Stress and HPA Axis Changes

Pregnancy and childbirth are accompanied by major changes in the HPA axis function. During pregnancy, placental corticotropin-releasing hormone and rising cortisol progressively activate the maternal stress system, with cortisol levels peaking near delivery (47,48). Following childbirth, placental endocrine signaling is withdrawn, and the HPA axis gradually recalibrates toward its nonpregnant state. This transition creates a period of altered stress responsivity during early postpartum recovery.

At the same time, the postpartum period introduces numerous psychological stressors, including infant-care demands, breastfeeding difficulties, relationship strain, low social support, and financial adversity (49, 50, 51, 52). Consistent with this observation, psychosocial stress is among the most robust risk factors for PPD, and longitudinal studies indicate that perceived stress predicts postpartum depressive symptoms (53,54). Large perinatal studies specifically associate partner-related and financial stressors with increased postpartum depressive symptoms (55).

Stress may be particularly relevant because chronic activation of stress-responsive systems influences inflammatory signaling, reward processing, and dopamine-dependent motivation. Although evidence linking HPA axis dysregulation to PPD remains mixed, altered cortisol dynamics and impaired adrenocorticotropic hormone–cortisol coupling have been reported in women with PPD and anxiety symptoms (48,56). Experimental evidence outside the postpartum period further demonstrates that chronic stress can suppress mesolimbic dopamine function through prefrontal mechanisms: in rodents exposed to chronic mild stress, reduced ventral tegmental area (VTA) dopamine neuron population activity was reversed by inactivation of the infralimbic PFC (57). Thus, postpartum stress may interact with prefrontal-mesolimbic systems regulating motivation, although this mechanism remains to be demonstrated directly in PPD.

### Immune Adaptation Across Pregnancy and Postpartum

Pregnancy requires substantial immune adaptation. The maternal immune system must simultaneously tolerate the semiallogeneic fetus while maintaining protection against infection (58,59). Rather than representing generalized immunosuppression, pregnancy is characterized by dynamic immune shifts across gestation. Inflammatory processes support implantation, relative immune tolerance facilitates fetal development, and increased inflammatory activity emerges toward parturition (8,58,59).

Inflammatory activity generally increases during late pregnancy and delivery before declining postpartum as pregnancy-related immune adaptations begin to resolve (8,60,61). Longitudinal studies demonstrate increases in interleukin 6 (IL-6), tumor necrosis factor α (TNF-α), and IL-1β across pregnancy, followed by reductions toward early-pregnancy levels during the postpartum period (60). Similarly, proteomic analyses indicate that many immune-related markers decrease after delivery, although a subset remains elevated or increases during the postpartum recovery (61).

Growing evidence links dysregulated inflammatory signaling to perinatal depressive symptoms and postpartum mental illness (6,62, 63, 64). Systematic reviews have identified associations between inflammatory biomarkers and perinatal depression, whereas studies in postpartum populations suggest that immune activation and kynurenine pathway dysregulation may be particularly relevant in severe or suicidal forms of PPD (62,63).

Importantly, inflammatory mediators provide a biologically plausible route through which postpartum adaptations may influence neural systems involved in motivation. Cytokines such as IL-6, TNF-α, and IL-1β can signal peripheral immune activity to the brain (Box 1 and Figure 1). After pregnancy, immune adaptation continues throughout the postpartum period. Longitudinal studies suggest that inflammatory markers follow heterogeneous trajectories after childbirth, with some cytokines declining toward prepregnancy levels, whereas others remain elevated during postpartum recovery (60,61). Furthermore, systematic reviews indicate that associations between postpartum depressive symptoms and peripheral inflammatory biomarkers remain inconsistent (63,64). Consequently, it remains unclear whether PPD is characterized by sustained peripheral inflammation or whether inflammatory exposure during pregnancy and the early postpartum period initiates long-lasting neural changes that persist after peripheral immune markers have normalized.

Box 1 **Cytokines and Immune-to-Brain Signaling**

Cytokines are small signaling proteins released by immune and nonimmune cells that coordinate inflammatory responses throughout the body. Proinflammatory cytokines such as IL-6, IL-1α, IL-1β, and TNF-α, play essential roles in host defense, tissue repair, and immune communication. Under physiological conditions, cytokine production is tightly regulated and typically resolved after elimination of an infectious or inflammatory stimulus. However, persistent or excessive cytokine production may prolong immune-to-brain signaling, promoting neuroinflammatory responses.

Peripheral cytokines can influence the brain through several complementary pathways (Figure 1):

A. BBB-mediated transport

Some cytokines can cross the BBB through saturable transport mechanisms rather than passive diffusion. Experimental radiolabeled cytokine studies showed that IL-1α, IL-1β, and TNF-α can enter the brain from blood through regulated BBB transport systems (51, 52, 53). This route allows small amounts of circulating inflammatory signal to reach the CNS directly.

B. Endothelial signaling

Peripheral cytokines can also signal to the brain without crossing the BBB. IL-1β and IL-6 can activate receptors on brain endothelial cells, inducing cyclooxygenase/prostaglandin pathways and local prostaglandin E2 production, which then signals to neural circuits controlling sickness responses such as fever and reduced activity (54,55). In this pathway, the BBB functions as an immune-sensing interface rather than simply a barrier.

C. Circumventricular organs

Circumventricular organs are brain regions with fenestrated capillaries and a reduced BBB, allowing them to detect blood-borne inflammatory signals more directly. Experimental studies show that systemic IL-1β activates cells near blood-brain interfaces and that subfornical organ neurons respond directly to IL-1β (56,57). These regions can relay peripheral immune information to hypothalamic and limbic circuits.

D. Vagal afferents

Peripheral cytokines can also communicate with the brain through neural signaling. Experimental studies showed that subdiaphragmatic vagotomy attenuates brain cytokine responses and sickness-related effects after peripheral IL-1β modulates rat subfornical organ neurons as a result of activation of a nonselective cationic conductance, indicating that vagal afferents transmit inflammatory information from the body to the brainstem (56,57). This pathway does not require cytokines to enter the CNS directly.

BBB, blood-brain barrier; CNS, central nervous system; IL, interleukin; TNF-α, tumor necrosis factor α.

**Figure 1:**
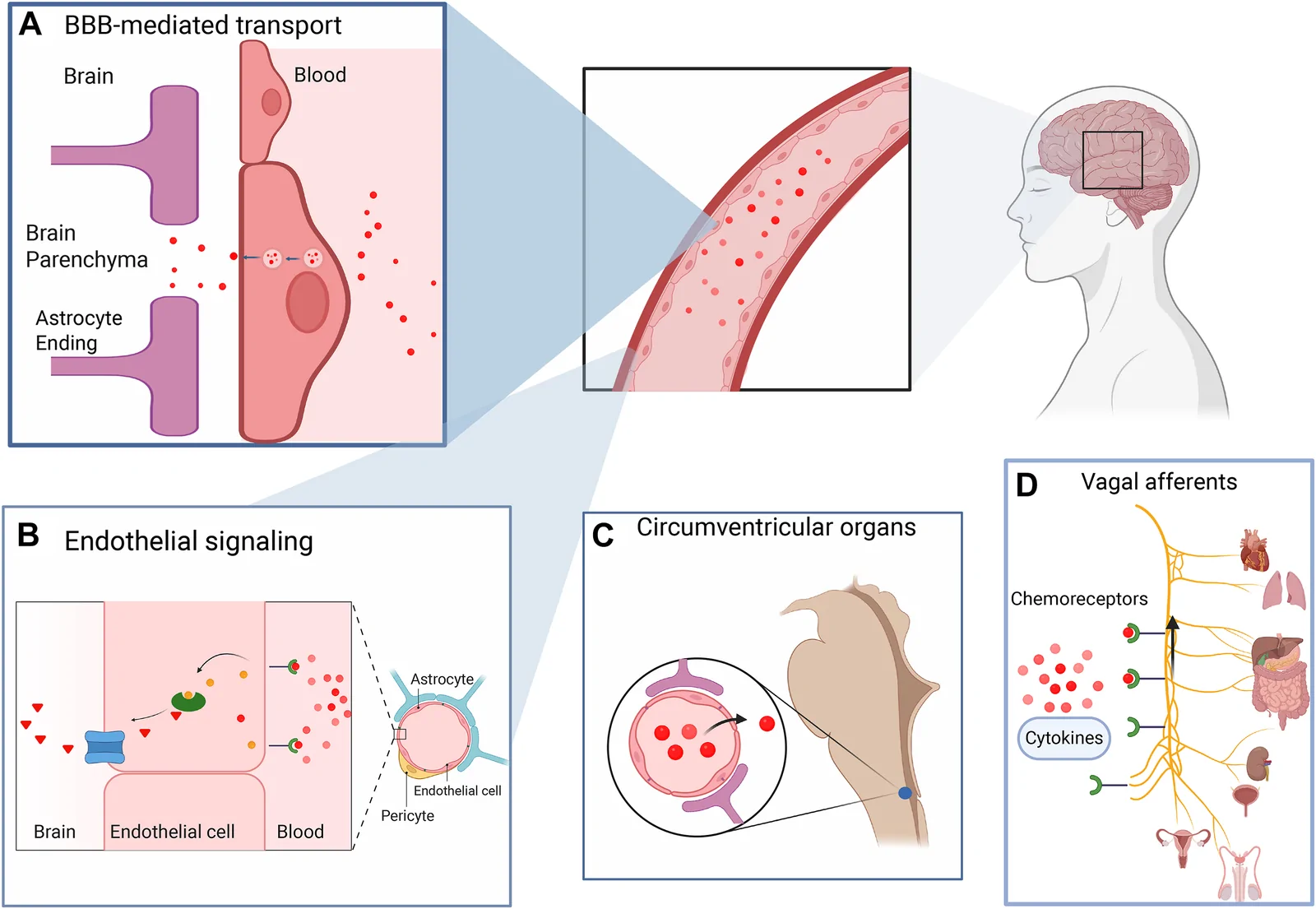
Pathways of peripheral immune-to-brain cytokine signaling. Schematic overview of the 4 primary mechanisms by which peripheral inflammatory signals communicate with the central nervous system. **(A)** BBB-mediated transport: Circulating cytokines (e.g., IL-1α, IL-1β, and tumor necrosis factor α) actively cross the intact BBB via vesicular transport systems to enter the interstitial space between astrocyte endfeet. **(B)** Endothelial signaling: Peripheral cytokines bind to receptors on brain endothelial cells without crossing the barrier, triggering intracellular signaling cascades (e.g., cyclooxygenase pathways) that release secondary messengers such as prostaglandin E2 into the brain parenchyma. **(C)** Circumventricular organs: Blood-borne cytokines gain direct entry into specialized brain regions characterized by leaky, fenestrated capillaries and a reduced BBB, allowing rapid interaction with local neural circuits. **(D)** Vagal afferents: Peripheral cytokines utilize neural pathways by activating receptors on vagal nerve terminals, which rapidly transmit inflammatory data from body organs directly to the brainstem without requiring physical entry of cytokines into the brain. BBB, blood-brain barrier; IL, interleukin.

This uncertainty is particularly relevant to the mechanistic models considered in the next section. Sleep disruption, stress, and hormonal adaptation may interact with immune signaling during a period in which the mesolimbic reward system is adapting to motherhood (65).

## Neuroinflammation, Dopamine Dysfunction, and Maternal Anhedonia

Anhedonia is increasingly recognized as a core symptom of PPD and may manifest as reduced motivation and impaired engagement in maternal behaviors (66,67). Growing evidence suggests that inflammation can influence neural systems supporting motivation (15,19,68). Experimental inflammatory challenges in nonpregnant adults reduce effort expenditure and reward responsiveness, whereas studies in nonperinatal depressed populations associate elevated inflammatory markers with disrupted connectivity within corticostriatal and mesolimbic circuits (20,69,70).

Evidence from general motivational neuroscience identifies NAc dopamine as a central regulator of motivated behavior (71, 72, 73). In rodent models, reductions in NAc dopamine decrease willingness to work for a reward despite preserved reward consumption. Restoration of dopamine increases reward-seeking behavior and effort (15,68,74). These observations have led to the view that motivational aspects of anhedonia may arise, at least in part, from the disruption of dopamine-dependent signaling within VS circuitry.

Animal models of PPD consistently demonstrate reduced activity of mesolimbic dopamine systems accompanied by impaired maternal motivation and anhedonia-like behavior (75, 76, 77). Human neuroimaging studies similarly report blunted responses to infant-related cues within the VS in mothers experiencing depressive symptoms (78,79). Together, these findings suggest that diverse postpartum biological disturbances may converge on neural systems regulating dopamine-dependent motivation.

An important unanswered question is how inflammation disrupts dopamine-dependent motivation. One possibility is that inflammation may act directly within the NAc, disrupting local mechanisms that regulate dopamine release. Alternatively, inflammatory signaling may impair distributed corticostriatal networks involved in cognitive control, reward valuation, and motivational regulation. Distinguishing between local VS mechanisms and network-level dysfunction may provide a useful framework for understanding how neuroinflammation contributes to maternal anhedonia.

### Local VS Mechanisms of Dopamine Dysfunction

Inflammatory processes may directly disrupt dopamine signaling in the NAc itself. Dopamine release within the NAc is determined not only by the firing of dopaminergic VTA neurons but also by local NAc microcircuit mechanisms. In particular, cholinergic interneurons (CINs) regulate dopamine release through acetylcholine-mediated activation of nicotinic acetylcholine receptors on dopaminergic axons (15,80, 81, 82).

Despite their small numbers, CINs exert powerful control over dopamine release. Acetylcholine activates predominantly β_2_-containing nicotinic receptors on dopamine varicosities, triggering dopamine release (80, 81, 82). Importantly, synchronized CIN activity can evoke dopamine release locally, even without ascending dopaminergic VTA neurons firing, highlighting the importance of VS microcircuits in regulating motivational behavior (81). CINs contribute to motivation through regulating dopamine release, which enables reward-directed behavior. In behaving rats, suppression of accumbal CIN activity reduced motivated reward seeking, whereas CIN activation promoted dopamine release and increased motivation, producing effects comparable to direct manipulation of dopamine signaling (83).

One potential pathway linking inflammation to local dopamine dysfunction involves activation of the kynurenine pathway. Peripheral inflammatory mediators such as IL-6, TNF-α, and interferon gamma stimulate IDO1, increasing the conversion of tryptophan to kynurenine. Kynurenine readily crosses the blood-brain barrier and is subsequently metabolized by astrocytes to kynurenic acid (KYNA), an endogenous neuromodulator with inhibitory effects on cholinergic neurotransmission (84, 85, 86). Experimental studies have demonstrated that elevated KYNA levels reduce extracellular dopamine concentrations in the striatum, whereas pharmacological inhibition of KYNA synthesis produces marked increases in dopamine release (87,88).

KYNA is a well-established antagonist of α7 nicotinic acetylcholine receptors and has been shown to attenuate cholinergic facilitation of dopamine release within the striatum (86,89). In animal models, elevations in brain kynurenine or KYNA blunt NAc dopamine responses to rewarding stimuli, including ethanol and psychostimulants (89,90). Notably, restoration of α7 nicotinic receptor signaling partially reverses these dopamine deficits, supporting a mechanistic role for impaired cholinergic regulation of dopamine transmission (90). Although direct evidence linking KYNA to β_2_-containing nicotinic receptors located on dopamine varicosities remains limited, these findings collectively suggest that inflammation-induced alterations in cholinergic signaling may reduce the effectiveness of CIN-mediated dopamine release (Figure 2).

**Figure 2:**
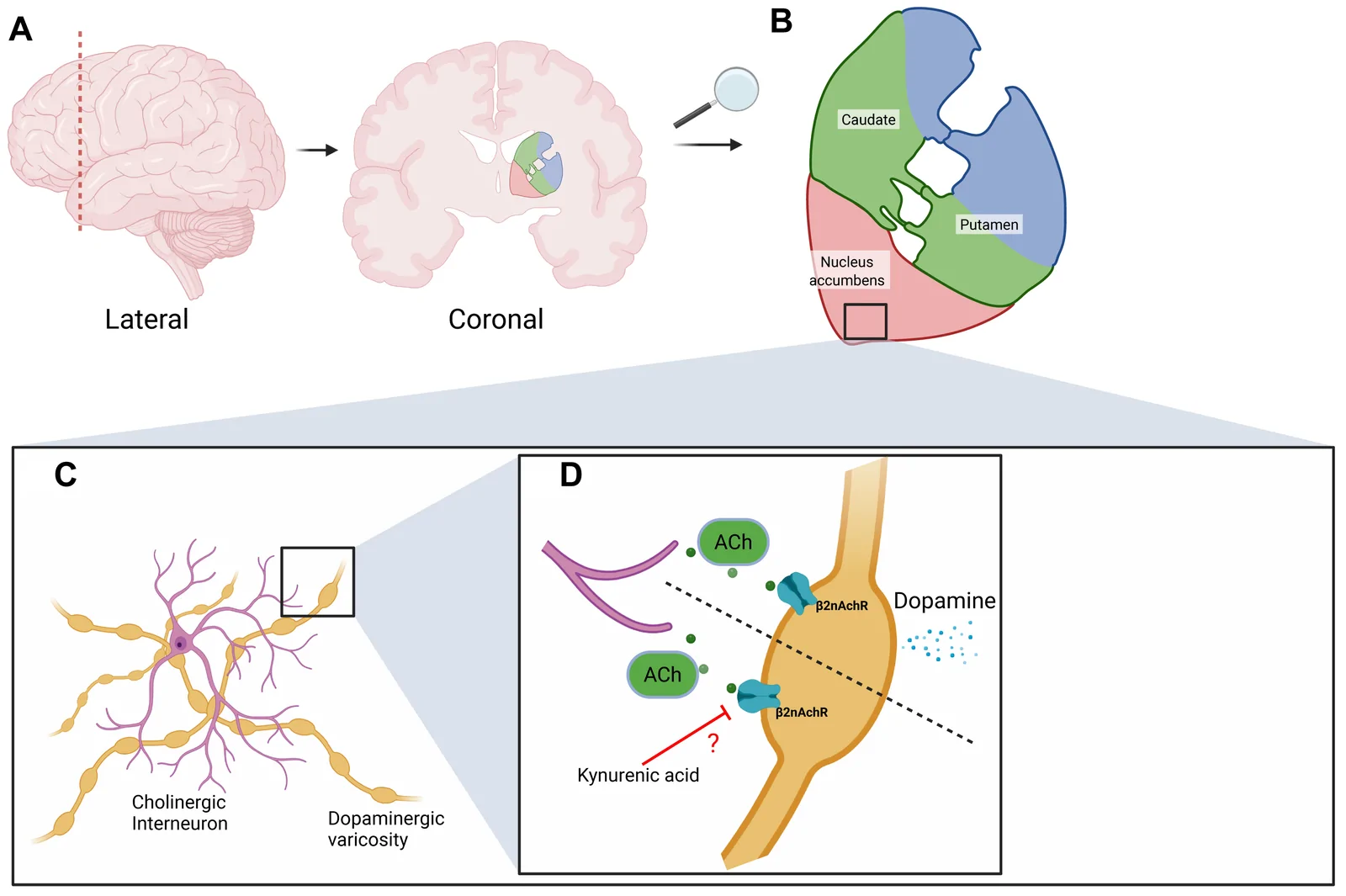
Local ventral striatal mechanisms. **(A)** Anatomical localization of the human striatum. Whole-brain perspective with a vertical red dashed line indicating the sagittal reference and a coronal brain slice perspective indicating the position of the striatum deep within the cerebral hemisphere (highlighted). **(B)** Striatal subdivisions in coronal view. Magnified coronal section detailing the anatomical distinctness of the caudate (green), putamen (blue), and nucleus accumbens (pink, a key component of the VS), showing their respective relative positions. **(C)** Local striatal microcircuit. Simplified representation of a large, dendritic cholinergic interneuron (purple) interacting with a dopaminergic varicosity (yellow). The region boxed indicates the cellular microenvironment for synaptic regulation. **(D)** Putative mechanism of neurotransmitter inhibition. Detail view of synaptic modulation: Kynurenic acid acts as an antagonist on cholinergic nicotinic receptors (nAChRs), which may disrupt normal neurotransmission by blocking the facilitating effects of Ach on local dopamine release. This suggests a potential localized mechanism for reduced motivational signals in the VS. [Parts of the illustration modified from (124,125). (Figure created in BioRender.)] ACh, acetylcholine; nAChR, nicotinic acetylcholine receptor; VS, ventral striatum.

Together, these findings support a model in which inflammatory processes may directly impair dopamine transmission in the VS through local circuit mechanisms. By disrupting cholinergic regulation of dopamine release, inflammation may reduce the motivational impact of rewards and contribute to effort-related aspects of anhedonia.

### Corticostriatal Mechanisms and Motivational Dysfunction

An alternative to direct NAc microcircuit dysfunction is that inflammation disrupts a broader corticostriatal network regulating motivation. The vmPFC is anatomically heterogeneous and includes medial and subgenual territories encompassing pregenual ACC (pgACC)/area 32 (A32) and sgACC/area 25 (A25) (91). Thus, the dlPFC-pgACC-sgACC-NAc sequence considered here represents a more anatomically specific route within the broader dlPFC-vmPFC-VS network. In the proposed model, inflammation-related dlPFC dysfunction weakens the regulation of A32/A25 circuitry, allowing abnormal A25 output to disrupt downstream NAc-dependent motivational processing.

Primate evidence provides increasingly strong support for the upstream portion of this model. Anatomical studies demonstrate serial pathways from the dlPFC regions through pgACC/A32 toward A25 (92,93). Human studies in nonperinatal depression show abnormal dlPFC-sgACC connectivity and demonstrate that dlPFC stimulation can modulate and suppress sgACC activity (94, 95, 96, 97, 98, 99, 100). Critically, recent causal experiments in marmosets extend these findings: inactivation of dlPFC A46 reduced effortful responding for reward, and pathway-specific inhibition demonstrated that this motivational deficit depended on the A46→A32 projection. Ketamine administration directly into A25 prevented the motivational deficit produced by A46→A32 inactivation, functionally linking A46, A32, and A25 within the same motivational network (101). Because stress and inflammatory signaling impair dlPFC neuronal function and network stability (Box 2 and Figure 3) (102, 103, 104), these findings provide a plausible route whereby inflammation-related dlPFC dysfunction could propagate through A32 toward A25.

Box 2 **Stress and Inflammation Disrupt dlPFC Function**

Peripheral inflammatory signals can reach the brain through several pathways described in Box 1, where they can initiate or amplify neuroinflammatory responses. Cytokine signaling within the CNS can activate microglia and alter astrocyte function, creating a proinflammatory neural environment. Activated microglia release proinflammatory cytokines, reactive oxygen species, and glutamate, while astrocytes exhibit reduced glutamate uptake. These changes shift the cortical microenvironment toward excitatory instability and impair the precise synaptic regulation required for higher-order cognitive functions. Because the dlPFC depends on highly specialized recurrent excitatory networks, it appears particularly vulnerable to inflammatory and stress-related insults (92).

Layer III pyramidal “delay cells” in the dlPFC maintain persistent firing that supports working memory, goal maintenance, and cognitive control. This activity relies on NMDA receptor signaling, particularly GluN2B-containing receptors, α7 nicotinic receptor–mediated modulation, and intracellular calcium amplification through the cAMP-PKA-Ca^2+^ signaling cascade (92,93). Although highly efficient, this circuitry is metabolically demanding and sensitive to stress and inflammation. Proinflammatory cytokines induce IDO1, shifting tryptophan metabolism toward kynurenine production (Figure 3). Kynurenine is subsequently converted—primarily by astrocytes—into KYNA, which antagonizes NMDA receptors and has been proposed to interfere with α7 nicotinic receptor signaling (103, 104, 105). Recent primate studies demonstrate that elevated KYNA markedly reduces dlPFC delay-cell firing and impairs working memory performance (105).

Neuroinflammation simultaneously disrupts intracellular regulatory mechanisms within dlPFC neurons. Activated microglia increase expression of GCPII, reducing availability of NAAG and weakening mGluR3-mediated inhibition of cAMP production (94). Combined with inflammatory alterations in calbindin signaling and phosphodiesterase localization, this loss of regulation permits excessive cAMP-Ca^2+^ activity, opening potassium channels such as HCN, KCNQ, and SK channels and thereby suppressing persistent neuronal firing (92,94).

Collectively, these receptor-level, synaptic, and intracellular disturbances weaken dlPFC function and reduce its ability to regulate downstream affective and motivational circuitry. Through diminished top-down control over sgACC and mesolimbic dopamine pathways, stress-related neuroinflammation may contribute to impaired motivation, reduced reward responsiveness, and the emergence of anhedonia.

cAMP, cyclic adenosine monophosphate; CNS, central nervous system; dlPFC, dorsolateral prefrontal cortex; GCPII, glutamate carboxypeptidase II; KYNA, kynurenic acid; mGluR, metabotropic glutamate receptor; NAAG, *N*-acetylaspartylglutamate; PKA, protein kinase A; sgACC, subgenual anterior cingulate cortex.

**Figure 3:**
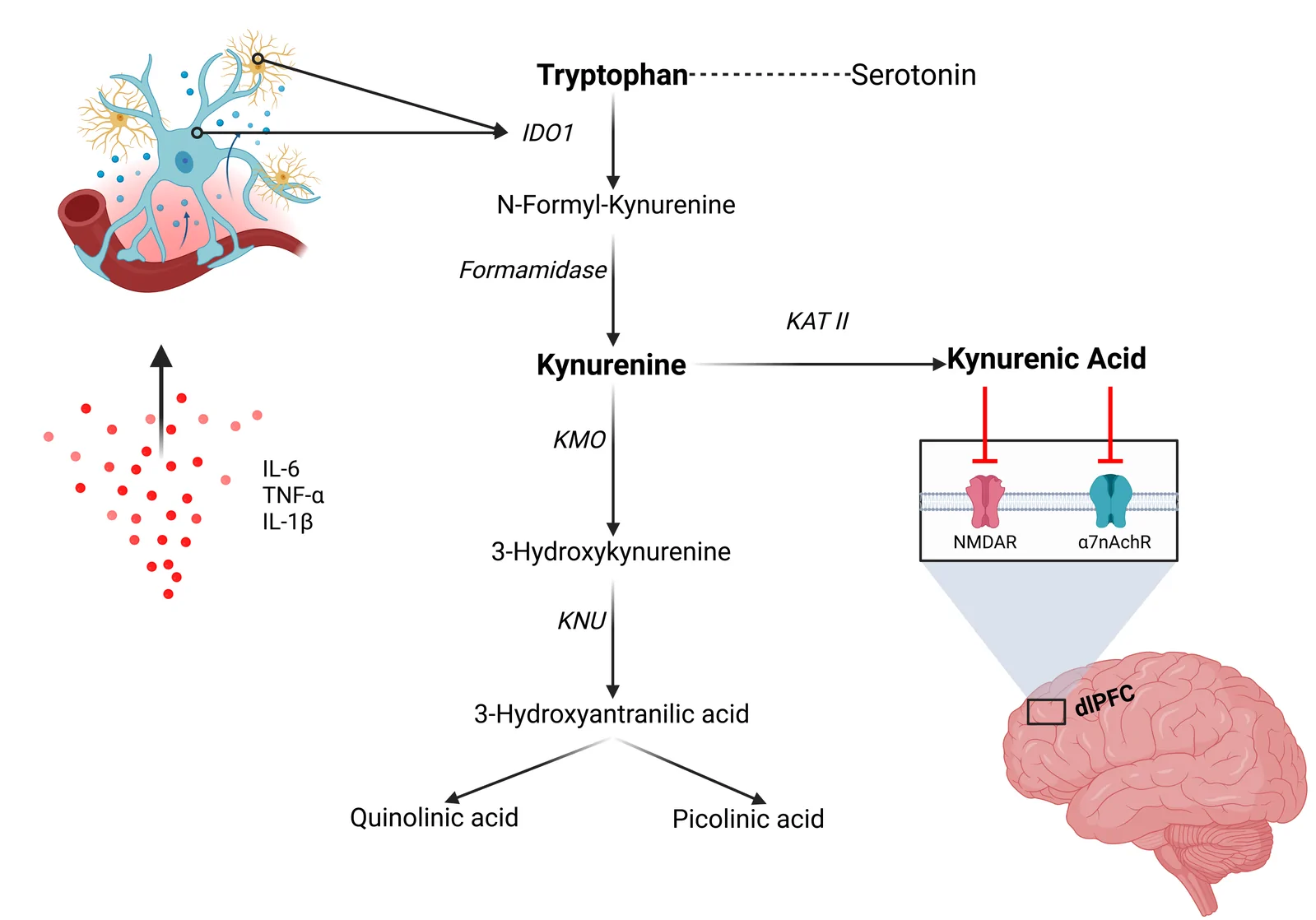
Inflammatory alteration of tryptophan-kynurenine metabolism and its impact on dlPFC circuitry. Peripheral proinflammatory cytokines (IL-6, TNF-α, and IL-1β) cross or signal through the blood-brain barrier to activate microglia and astrocytes within the central nervous system. This neuroinflammatory state drives the expression of IDO1, shifting tryptophan metabolism away from serotonin synthesis and toward the kynurenine pathway. Kynurenine is subsequently metabolized by astrocytes (via KAT II) into KYNA. Within the dlPFC, elevated KYNA acts as an antagonist at NMDAR and α7nAch receptor, disrupting the persistent delay-cell firing necessary for working memory and cognitive control. dlPFC, dorsolateral prefrontal cortex; IL, interleukin; KMO, kynurenine 3-monooxygenase; KYNA, kynurenic acid; nAChR, nicotinic acetylcholine receptor; NMDAR, NMDA receptor; TNF-α, tumor necrosis factor α.

Excessive sgACC/A25 activity may represent a critical downstream consequence of this disrupted regulation. sgACC hyperactivity is implicated in human depression, and abnormal A25 responses to positive incentives have been associated with anhedonia (105). In nonhuman primates, experimental A25 overactivation blunts reward anticipation and effort-related responding (106). Selective activation of the A25-NAc projection reduces behavioral and cardiovascular responses during reward anticipation without impairing reward consumption (107). Together, these studies support a candidate sequence in which impaired dlPFC function alters A32/A25 regulation, whereas excessive A25→NAc signaling disrupts motivational processing. However, dopamine was not measured in these primate experiments; therefore, whether this pathway produces motivational impairment by reducing NAc dopamine remains unknown.

The cellular mechanism through which excessive cortical input alters NAc function is similarly unresolved but may intersect with the local dopamine-regulating mechanisms described in Local VS Mechanisms of Dopamine Dysfunction. Cortical projections to the NAc are glutamatergic, and rodent studies demonstrate that medial prefrontal afferents can directly excite both medium spiny neurons and CINs in the NAc core (108). Because CINs locally regulate dopamine release through nicotinic receptors on dopamine axons (80, 81, 82, 83), abnormal cortical input could potentially alter local dopamine regulation without requiring a primary change in VTA dopamine-neuron firing. However, the available cellular evidence derives from rodent mPFC rather than primate A25/A32, and an A25/A32→NAc CIN→dopamine mechanism has not been demonstrated. It therefore represents a plausible point of convergence between the network and local mechanisms proposed in this review rather than an established pathway.

Human inflammation studies provide broader support for dysfunction of this network but generally examine its shorter vmPFC-VS component rather than the complete dlPFC-A32-A25-NAc sequence. In nonperinatal populations, higher peripheral inflammation is associated with reduced vmPFC-VS connectivity, broader vmPFC-centered abnormalities, altered sgACC-mesolimbic connectivity, and greater anhedonia or reduced reward responsiveness (71,109, 110, 111, 112). These observations are consistent with the roles of ventromedial prefrontal regions in reward valuation (113, 114, 115) and the dlPFC in cognitive control and maintenance of goal-directed behavior (116,117). Taken together, the evidence supports a candidate hierarchical model in which inflammation-sensitive dlPFC dysfunction propagates through A32/A25 circuitry toward the NAc, where abnormal cortical input may ultimately interfere with dopamine-dependent motivational processing. The individual components of this pathway are supported across human, nonhuman primate, and rodent studies; however, the complete causal sequence—and particularly its relevance to PPD—remains to be demonstrated.

## Neuroimaging Evidence Supporting Reward Dysfunction in PPD

Neuroimaging studies provide converging evidence that PPD is associated with abnormalities in brain regions involved in motivation, reward processing, and emotional regulation. Functional magnetic resonance imaging (fMRI) studies consistently demonstrate reduced neural responses to infant-related cues within the VS, mPFC, ACC, and amygdala, suggesting diminished motivational salience of rewarding social stimuli (13,78,79,118). These findings are consistent with behavioral observations of reduced maternal motivation, impaired responsiveness to infant cues, and disrupted maternal-infant bonding in women with PPD.

Among cortical regions, the dlPFC has emerged as one of the most consistently implicated structures. Structural MRI studies have reported alterations in dlPFC volume and cortical thickness in women with PPD, whereas functional imaging studies have demonstrated abnormal dlPFC activation and connectivity with networks involved in cognitive control and emotional regulation (119, 120, 121). Because the dlPFC is highly sensitive to stress and inflammatory signaling and occupies a central position within corticostriatal networks, these findings provide indirect support for the hypothesis that neuroinflammation may contribute to motivational deficits through disruption of distributed prefrontal circuitry.

However, current neuroimaging findings do not definitively distinguish between the 2 mechanistic models proposed in this review. On one hand, abnormalities involving the dlPFC, ACC, vmPFC, and VS are broadly consistent with a corticostriatal model in which inflammation disrupts large-scale networks regulating reward valuation and motivation (71,94,95,100,109, 110, 111). In support of this view, inflammatory markers have been associated with reduced connectivity within vmPFC–ventral striatal circuitry, altered sgACC activity, and impaired reward responsiveness in depression. Furthermore, recent studies demonstrate that dlPFC stimulation can directly modulate sgACC activity, providing a physiological pathway through which prefrontal dysfunction could influence motivational processing (94, 95, 96,100).

On the other hand, neuroimaging evidence for local ventral striatal dysfunction remains comparatively limited. Reduced VS activation is among the most replicated findings in studies of anhedonia and maternal reward processing (78,79,118), but conventional MRI cannot determine whether these abnormalities arise from altered corticostriatal inputs or from local disruptions of dopamine-regulating microcircuits within the NAc. Consequently, current neuroimaging evidence is more consistent with network-level dysfunction than with a purely local VS mechanism, although existing methods cannot exclude the latter possibility.

A major limitation of conventional MRI is its inability to directly measure dopamine transmission or neuroinflammatory activity. Positron emission tomography (PET) imaging can partially overcome this limitation by quantifying dopamine synthesis, receptor availability, transporter binding, dopamine release, and neuroinflammatory markers such as translocator protein expression (122,123). Integration of PET with structural and fMRI may therefore provide a means of testing competing mechanistic models. The corticostriatal model predicts that inflammatory abnormalities should be associated with dysfunction of prefrontal and cingulate networks preceding or accompanying ventral striatal dopaminergic abnormalities. In contrast, the local ventral striatal model predicts that inflammatory and dopaminergic abnormalities should be detectable within the VS even in the absence of major cortical dysfunction. Future multimodal PET-MRI studies incorporating inflammatory biomarkers, measures of motivation, and longitudinal designs may therefore help determine whether maternal anhedonia arises primarily from distributed network dysfunction, local ventral striatal pathology, or interactions between both mechanisms.

### Limitations and Future Directions

Several limitations currently constrain the interpretation of the neuroimaging literature. First, relatively few imaging studies have specifically examined PPD, and many conclusions rely on major depressive disorder. Second, most available studies employ cross-sectional designs, limiting causal inference. Third, dopamine dysfunction is generally inferred from neural activity and connectivity measures rather than directly measured dopamine levels. Fourth, sample sizes remain modest, and imaging methodologies vary substantially across studies.

An additional unresolved question concerns the directionality of these relationships. The models discussed in this review assume that abnormalities within corticostriatal networks contribute to downstream dysfunction of VS dopamine signaling. However, an alternative possibility is that local disturbances in VS dopamine transmission may themselves impair the recruitment of broader motivational and cognitive-control networks. Because motivated behavior emerges through reciprocal interactions between mesolimbic and cortical systems, current evidence is insufficient to determine whether dopamine dysfunction is primarily a consequence of network dysregulation or a driver of network dysfunction.

Future multimodal studies integrating inflammatory biomarkers, neuroimaging, and behavioral measures of motivation will be necessary to determine whether motivational dysfunction emerges primarily from local ventral reward alterations, distributed circuit dysregulation, or an interaction between both mechanisms.

## Conclusions

Maternal anhedonia is increasingly recognized as a clinically important component of PPD; however, its underlying neurobiology remains incompletely understood. The postpartum period combines sleep disruption, hormonal withdrawal, psychosocial stress, and a distinctive immune trajectory during a window in which the mesolimbic reward system is itself reorganizing around motherhood. Neuroinflammation offers a biologically plausible route by which these adaptations converge on the dopamine-dependent circuitry that supports maternal motivation, acting either locally within NAc microcircuits or through distributed corticostriatal networks involving the dlPFC, pgACC, sgACC, and vmPFC.

The corticostriatal pathway is currently supported by more extensive circuit-level causal evidence than the local pathway, though neither has been demonstrated directly in PPD. Primate circuit-tracing and causal manipulation studies place the dlPFC, pregenual A32, and subgenual A25 in series, and recent chemogenetic and lesion work shows that disrupting this sequence is sufficient to blunt reward-directed motivation. Human neuroimaging in nonperinatal depression independently shows that inflammatory markers track reduced vmPFC–ventral striatal connectivity and altered sgACC activity and that dlPFC stimulation can modulate sgACC output directly. Taken together, this explains why the existing PPD and depression neuroimaging literature is more consistent with network-level dysfunction than with a purely local ventral striatal mechanism—though conventional imaging cannot exclude a local contribution, and the inflammation-connectivity evidence itself comes almost entirely from nonperinatal samples rather than from postpartum women.

This should temper rather than settle the conclusion. Reduced ventral striatal response to reward and to infant cues remains among the most consistently replicated findings in PPD itself, and the local mechanisms proposed—CIN regulation of dopamine release and its potential disruption by kynurenine pathway metabolites—offer a physiologically direct route to reduced NAc dopamine release that has simply not yet been tested with the tools available. The 2 pathways may also not be fully separable at the circuit level: cortical projections to the NAc synapse onto the same CINs implicated in the local model, so corticostriatal and local dysfunction could in principle converge on a shared cellular mechanism rather than representing 2 independent tenets of an anhedonia model.

A further limitation is directionality: whether corticostriatal dysfunction drives downstream dopaminergic impairment or whether a primary striatal disturbance instead limits the recruitment of cortical control and valuation networks cannot be determined from the cross-sectional evidence available. Multimodal PET-MRI, capable of directly indexing dopamine synthesis, receptor availability, and neuroinflammation alongside functional connectivity, is the most direct means of adjudicating between these possibilities, and applying such designs longitudinally across the peripartum period—when the relevant inflammatory and hormonal changes occur on a known, trackable timeline—would allow researchers to test whether striatal or cortical abnormalities emerge first, rather than inferring sequence after the fact.

We propose that maternal anhedonia arises when postpartum inflammatory signaling disrupts the motivational salience of infant-related and other rewarding stimuli, most plausibly through some combination of corticostriatal network dysfunction and local striatal dopamine dysregulation, the relative contributions of which remain to be established. Clarifying this balance would help identify whether biologically informed interventions for maternal anhedonia are better targeted at the level of prefrontal-cingulate circuitry—for example, through neuromodulation—or at striatal neurotransmitter and inflammatory mechanisms directly and would help determine whether these targets differ across individuals or across the postpartum timeline.
